# The financial burden experienced by families during NICU hospitalization and after discharge: A single center, survey-based study

**DOI:** 10.1007/s00431-023-05352-y

**Published:** 2023-12-01

**Authors:** Carmine Vincenzo Lambiase, Giuseppina Mansi, Serena Salomè, Maria Laura Conelli, Maria Vendemmia, Maria Clelia Zurlo, Francesco Raimondi, Letizia Capasso

**Affiliations:** 1 Division of Neonatology, Department of Translational Medical Sciences, University of Naples Federico II, Via Sergio Pansini 5, 80131 Naples, Italy; 2 Department of Humanities, University of Naples Federico II, Via Porta di Massa 1, 80133 Naples, Italy; 3 Dynamic Psychology Laboratory, Department of Political Sciences, University of Naples Federico II, Naples, Italy

**Keywords:** Financial burden, NICU graduate infants, Parents, NICU

## Abstract

To investigate a broad array of costs and perceived financial burden (FB) faced by families of NICU graduates both during hospitalization and after discharge. Cross-sectional survey-based study design. A survey measuring socio-demographics, direct non-medical costs, indirect costs, social support and perceived FB was developed. One-hundred-twenty-two pairs of parents of NICU graduates participated in the study. Most of the families (87.7%) experienced FB due to NICU hospitalization. The median cost of visiting infant during NICU admission was 615 euros (range: 42,7320). FB correlated with cost for drugs (*ρ* = .271, *p* < .05, 95%CI:[.020, .490]), dietary supplement (*ρ* = .385, *p* < .05, CI:[.010, .665]), behavioral disorders (*ρ* =  −.186, *p* < .05, 95%CI:[−.356, −.003]), language delay (*ρ* = .243, *p* < .01, CI:[−.408, −.063]) and comorbidities (*ρ* =  −.206, *p* < .05, 95% CI:[−.374, −.024]). Transportation costs due to medical visits (*ρ* = .415, *p* < .01, 95% CI:[.239, .564]) and therapy sessions (*ρ* = .517, *p* < .05, CI:[.121, .771]) correlated with higher FB. Grandparents of the infant were the most frequent source of help (86.1%). Families having infants with adverse outcome experienced more hospitalizations after NICU discharge (*p* < .05) and higher FB (*p* < .01) than families with typically developing infant. Lack of government financial help was associated with higher perceived FB (CI:[1.117,29.127], *p* < .05).

*   Conclusions*: Our findings demonstrated that parents of NICU graduates experience high rates of FB, highlighting their sources (e.g., grandparents support) and difficulties (e.g., private therapy costs) through the lens of patient perspective. Our study promotes reflection on policies which should be adopted from the European health services that are similar to the Italian one to support NICU graduate families and reduce inequalities.

**Table:** 

**What is Known:** *• Families of NICU graduates face several kinds of costs during hospitalization and after discharge.*	
**What is New:** *• NICU hospitalization is a multifaceted event that impact financial burden experienced by families.* *• NICU graduate families whose infant had adverse outcome and felt lack of financial help from local policy makers experience higher rates of financial burden.*

**Supplementary Information:**

The online version contains supplementary material available at 10.1007/s00431-023-05352-y.

## Introduction

One in eight babies born each year will be needing special care [1]. A number of studies highlighted the financial burden (FB) experienced by families during NICU hospitalization [2]. Fewer studies investigated the costs faced by families after discharge, especially in Europe [3]. Despite that FB has been studied in patients and caregivers [2, 4], its definition is not clear yet [5]. The FB is typically described as one’s perceptions of the financial strain (i.e., the subjective pressures) and stress (i.e., objective economic challenges) [6, 7]. To our knowledge, no Italian studies examined the FB experienced by families of NICU graduate infants both during hospitalization and after discharge. Further, a systematic review [2] recently underlined the paucity of studies that takes into account direct medical costs (e.g., medical outpatient visits), non-medical costs (e.g., cost for transportation at medical outpatient visit) and indirect costs (e.g., loss of work due to specific event). To address both these gaps, we aimed to investigate a broad array of costs (i.e., direct and non-direct medical costs, indirect costs) and perceived FB of Italian families both during NICU hospitalization and after discharge.

## Materials and methods

This was a cross-sectional, survey-based study. Parents of NICU graduate infants were asked to fill in the survey during the routinely developmental follow-up visit from September 2022 to May 2023. Two research psychologists orally informed them about the study. The study protocol was approved by the Ethics Committee “Carlo Romano” of the University Federico II of Naples (protocol number 68/22). Written consent was obtained from all the participants to the study. Inclusion criteria were the following: being parent of an infant who joined our developmental follow-up program and fluency in Italian written form of at least one parent. Exclusion criteria were the following: infant did not survive after NICU discharge or enrollment in another developmental follow-up program.

### Measures

**Fig. 1:**
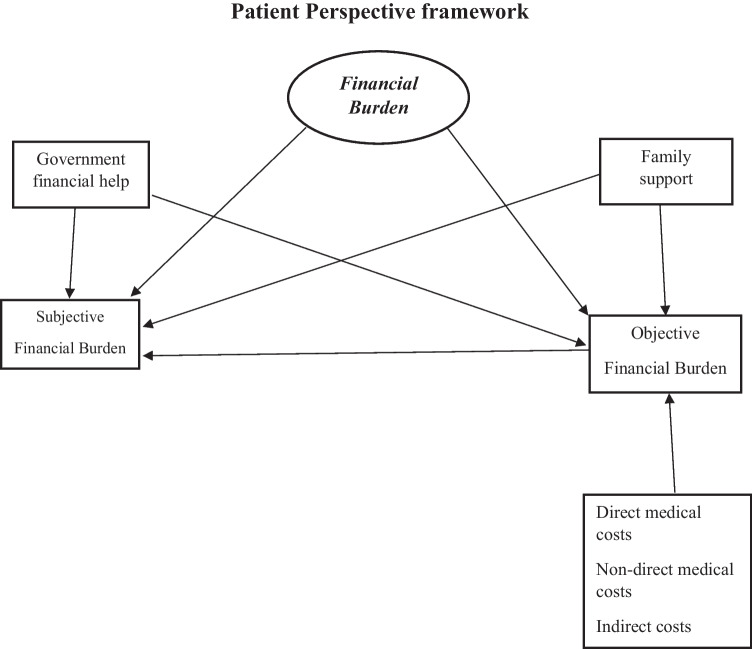
Financial burden in NICU graduate parents through the lens of patient perspective within the Italian context

We developed an ad hoc survey to investigate the FB experienced by families during NICU hospitalization and after discharge. Items were derived within a patient perspective framework and considering studies on financial burden in NICU parents [2, 3]. Both objective (i.e., effective costs) and subjective (i.e., individual perception) aspects of financial burden were considered [8]. Inputs from neonatologists (*n* = 2), NICU psychologists (*n* = 2) and one health care management professor were collected to adapt the content of the survey to the Italian NICU context and healthcare system (see Fig. 1 for a summary of the theoretical framework). The survey was preliminary reviewed and piloted for content and clarity by parents of NICU graduates referring to the Perinatal Infections center of our University Hospital. Items were dichotomous (presence vs. absence), on a 4-level Likert scale (ranging from “none” to “high”) or open questions in which parents could write down the amount of expenses faced (e.g., monthly outgoings for vitamins). The survey was divided as follows:

- Section 1 (13 items) socio-demographic information of both parents (e.g., age, job, education level) and characteristics of the family (e.g., presence of siblings, type of house held). Jobs were then divided according to the classification of the Italian National Institute of Statistics [9].
- Section 2 (11 items) NICU infant characteristics (e.g., gestational age, birthweight, type of pregnancy) and outgoings faced by families during NICU hospitalization. Total expenses during NICU hospitalization were then calculated for every family multiplying mean daily costs reported from parents and their length of stay in NICU (i.e., number of days).
- Section 3 (12 items) encompassed infant neurodevelopmental outcome based on the developmental follow-up visit at the time of survey administration, numbers and types of further hospitalizations after NICU discharge and types of financial exemptions received due to infant pathology.
- Section 4 (15 items) provided information regarding infant need for special aids and habilitation therapy (e.g., physiotherapy, speech therapy) and the related expenses faced by families.
- Section 5 (8 items) informed about family monthly specific expenses (e.g., vitamins, drugs, dietary supplement) and costs related to routinely and specialized medical check-ups and transportation costs.
- Section 6 (7 items) regards occupational changes during NICU hospitalization and work leave needed for issues related to the infant’s health after discharge.
- Section 7 (7 items) investigated the source of help received (e.g., grandparents, babysitter) type of help provided (e.g., economic help, handling other children and house) and the financial aids given from the government. Based on previous studies [10, 11], perceived FB experienced by families related to NICU hospitalization and after discharge was assessed using a single item created for the current study*.* This item was rated on a 4 point-Likert scale (1 = Not at all; 2 = Little; 3 = Moderate; 4 = High).

The questionnaire is provided as Supplementary information.

### Data analysis

Data analysis was performed using SPSS v.28 software (IBM). Quantitative variables were expressed as mean ± standard deviation (sd) and categorical variables as frequency and percentages. Chi-square test was used to examine associations among nominal variables. Wilcoxon test was run to examine perceived FB among families who had an infant with normal vs. adverse outcome and families who experienced financial help from the government vs. those who did not perceive it. Kruskal-Wallis test with Bonferroni correction for multiple comparisons was run to test differences among multiple groups stratified by gestational age, birthweight and COVID-19 pandemic periods. Spearman rho correlation was run to detect variables correlated with the FB experienced by families. Multivariable binary logistic regression to determine factors associated with the perception of family FB was run.

## Results

### NICU hospitalization expenses

A total of 125 pairs of parents were approached during the routinely developmental follow-up visit. Two couples (1.6%) refused to participate due to lack of time and one couple (0.8%) did not fully complete the survey due to lack of fluency in Italian written form. The final sample included 122 pairs of parents (97.6%). Socio-demographic information of mothers and fathers are shown in Table 1. Half of both mothers and fathers graduated high-school. A large number of mothers were classified within the category “Unskilled professions” while most part of fathers belonged to the “Artisans, specialized workers and farmers” category.

**Table 1:** Socio-demographic information of mothers (*n* = 122) and fathers (*n* = 120)

	**Mother %(n)**	**Father %(n)**
**Age**		
Mean(SD)	35.09(5.44)	38.68(6.56)
median(range)	35 (15–51)	39(19–66)
**Nationality**		
Italian	95,9 % (117)	96,7 % (118)
**Education**		
< High school	21.3 % (26)	29.2 % (35)
High school	48.4 % (59)	51.7 % (62)
University degree	30.3 % (37)	19.2 % (23)
**Job category**		
Legislators, Entrepreneurs and Highly skilled professions	13.2 % (16)	12.5 % (15)
Technical professions	12.3 % (15)	15 % (18)
Qualified professions in commercial activities and services and executive professions in office work	17.2 % (21)	25 % (30)
Artisans, skilled and not skilled workers, farmers and vehicle drivers	4.9 % (6)	34.2 % (41)
Unskilled professions	50.8 % (62)	9.2 % (11)
Armed forces	1.6 % (2)	4.2 % (5)

A large amount of families had their own house as compared to families who lived in a house for rent or at parent house. Detailed family characteristics can be found in Table 2.

**Table 2:** Family characteristics

		**%(*n*)**
Other children (number)	1	41.0% (50)
	> 1	13.1% (16)
Type of house	Owned house	57.4% (70)
	House for rent	30.3% (37)
	Living at family member's house	12.3% (15)
Residence	Naples and its district	74.6% (91)
	Other districts	25.4% (31)

During NICU hospitalization, 36.1% of mothers had to leave their job as compared to 20.5% of fathers. In total, 9.8% of parents were fired during NICU hospitalization (mothers = 4.9%; fathers = 4.9%).

Characteristics of the infants involved in the study are reported in Table 3. Median (range) length of stay in NICU was 50 days (5, 365). Median family daily expenses during NICU hospitalization were 15 euros (1.40, 60) encompassing travel expenses to NICU and personal expenses when needed (i.e., meals). The overall median cost of visiting infant during NICU hospitalization was 615 euros (42, 7320). A decreasing trend (*p* = 0.051) during COVID-19 pandemic (median = 495, range = 42, 3000) as compared to the pre-pandemic period (median = 825 euros, range = 90, 7320) was observed. Median costs after COVID-19 period raised to 600 euros (95, 4080). Mean costs of NICU hospitalization according to prematurity rate and birthweight category are shown in Table 4.

**Table 3:** Characteristics of the infants enrolled

**Infants (*n*** **= 145)**	**%(*n*)**
**Sex**	
Female	41.8% (51)
**Type of pregnancy**	
Natural conception	86.9% (106)
**Twin delivery**	19.7% (25)
**Number of twins** 2 > 2	13.1% (16) 3.3% (4)
**Prematurity rate** Mean(SD) Range	31.23 (4.08) 22.50–42
Extremely preterm Very preterm Moderate preterm Late preterm Term	13.9% (17) 45.9% (56) 22.1% (27) 6.6% (8) 11.5% (14)
**Birthweight category** Mean(SD) Range	1461.8 (812.73) 400–4100
< 1000 1000–1499 1500–2500 > 2500	27.9% (34) 43.4% (53) 17.2% (21) 11.5% (14)

*SD *standard deviation

**Table 4:** Total NICU hospitalization expenses (in euros) stratified by prematurity rate and birthweight category

	**Mean (SD)**
**Prematurity rate**	
Extremely preterm infants	1637.1 (1817.8)
Very preterm infants	1175 (1001.3)
Moderate preterm infants	705.9 (580.3)
Late preterm infants	662.1 (453.8)
Term infants	284.4 (181.8)
**Birthweight category**	
< 1000	1729.8 (1482.4)
1000–1499	759.9 (495.9)
1500–2499	851.9 (985.4)
≥ 2500	241.6 (124.3)

Differences in total expenses among groups stratified by gestational age were found (H(4) = 24.174, η^2^ = 0.218, *p* < 0.001). In particular, parents of very preterm (mean rank = 65.83, *p* < 0.001) and extremely preterm infants (mean rank = 71.73, *p* < 0.001) had higher expenses as compared to full term infants (mean rank = 22.50). Those differences were kept after Bonferroni correction. Statistically significant differences were found according to birthweight category (H(3) = 33.081, η^2^ = 0.298, *p* < 0.001). Parents of ELBW infants (mean rank = 78.48) faced higher costs due to NICU hospitalization as compared to the other birthweight categories. Parents of LBW (mean rank = 49.55, *p* = 0.007) and VLBW (mean rank = 55.52, *p* < 0.001) infants experienced higher costs as compared to parents of > 2500 g infants (mean rank = 18.38). No differences were found among LBW and VLBW infants (*p* = 0.490). Those differences survived after Bonferroni correction.

### Expenses after NICU discharge

Forty-five percent of infants had health or development related issue and 21.3% of them had at least one comorbidity at the time of participation. Detailed infant developmental outcomes can be found in Table 5. Twenty-seven percent went for at least one new hospitalization after NICU discharge and 46.7% went for medical day hospital (18% within our hospital). Almost all the infants (94.2%) had outpatient checkups (35.2% of them within our hospital) other than developmental follow-up visits. Number of medical checkups per year were assessed according to infant age at the time of administration of the survey (0–12 months = 35.24%; 13–24 months = 16.39%; ≥ 25 months = 48.36%). Almost the whole sample (97.67%) went for at least one medical visit within the first year after discharge. During the same period, most part of families (69.76%) went for more than 3 medical checkups per year, frequency rate decreased to 60% within the second year of life. At 25 months of age, 69.48% of families went for more than one medical checkup per year and 49.15% for more than 3 medical checkups. Twenty-one percent of families had exemptions due to low income while 14.8% of them received financial support for the pathology of the infant.

**Table 5:** Infant developmental outcomes

**Outcome**	**%(*n*)**
Normal outcome	56.5 (69)
Assisted ventilation	1.6 (2)
Plagiocephaly	2.5 (3)
Cerebral palsy	3.3 (4)
Growth deficits and diet issues	14 (17)
Intellectual disability	3.3 (4)
Autism spectrum disorder	1.6 (2)
Behavioral disorder	8.2 (10)
Global delay of psychomotor development	10.7 (13)
Language delay	12.3 (15)
Retinopathy of prematurity	5.7 (7)
Inguinal hernia	1.6 (2)
Heart disease	3.3 (4)
Deafness	1.6 (2)
Muscle tone anomalies	18 (22)
Broncho dysplasia	11.5 (14)

Most parents indicated that they needed less than 1 month (45%) of out-of-work days due to their own infant health-related issues while 18.5% of parents did not need out-of-work days as at least one parent did not work. A number of families needed 1 month (10.7%) free from work to take care of their infant or more than 1 month (29.5%) per year. Out-of-work days were not paid as were superior to the contract rules for 28% of families.

Special aids were used by 14.8% of families. Among them, 63.63% paid for special aids with expenses ranging from 20 to 40 euros (median = 27.50). Infants who needed physiotherapy (18.9%) started the treatment at median age (range) = 7 (3, 48) months. Families who paid for physiotherapy (52.17%) faced a cost of median (range) = 160 (120, 400) euros per month. Infants who needed habilitation therapy were 19.7%. Among them, 73.91% needed three therapies simultaneously and 17.39% needed two therapies. The most practiced therapies were speech (91.3%) and psychomotor therapy (86.95%). Habilitation therapy was started at median (range) = 24 (6, 84) months. Seventy-one percent of families paid for habilitation therapies. Costs for therapy sessions was median (range) = 300 (160, 600) per month while transportation at therapies reached median = 100 (2.40, 400) euros. Overall, 8.19% of infants started physiotherapy within the first year of life and at least one habilitation therapy within the following 2 years. Further expenses faced by families after discharge can be found in Table 6.

**Table 6:** Outgoings after NICU discharge (euro)

**Monthly costs after discharge (*n*)**	Mean (SD)	Median (range)
Lab exams (17)	104.12 (122.00)	50.00 (5–500)
Vitamins (95)	37.57 (24.06)	30.00 (7–150)
Drugs (64)	58.58 (64.52)	35.00 (7–300)
Milk and dietary supplement (29)	138.17 (100.87)	120.00 (20–450)
Medical visits (56)	81.65 (80.14)	67.50 (5–500)
Transportations at hospitals (107)^a^	105.10 (208.97)	40.00 (5–1300)
Costs out of region (3)^b^	2433.33 (2227.85)	1300.00

^a^within 1 year

^b^total costs

Parents felt supported by their own family (90.2%). Grandparents of the infant were the most frequent source of support (86.1%). Other helps found by families were nursery school (13.9%), babysitters (11.5%) and domestic workers (4.1%). Twenty-three percent of families needed more than one of the above-mentioned sources of help. Transportations (43.4%) and house handling (40.2%) were the most frequent helps received. Other children management was given to 29.5% of families. Twenty-nine percent of families received economic helps and 12.3% of parents received emotional support. Nine percent of families did not receive any kind of help and stated that their own partner was their only source of help. Nineteen percent of families indicated being supported by the government. Governmental economic helps frequently received were child support check (40.2%) and baby bonus (35.2%). Those checks were not helpful (50.8%) or only partially helpful (43.4%) for facing outgoings.

Overall, 87.7% of families experienced FB after NICU experience as impacting the whole family system. Sixty percent of families experienced moderate (50%) or high (10.7%) rate of FB due to NICU experience.

**Fig. 2:**
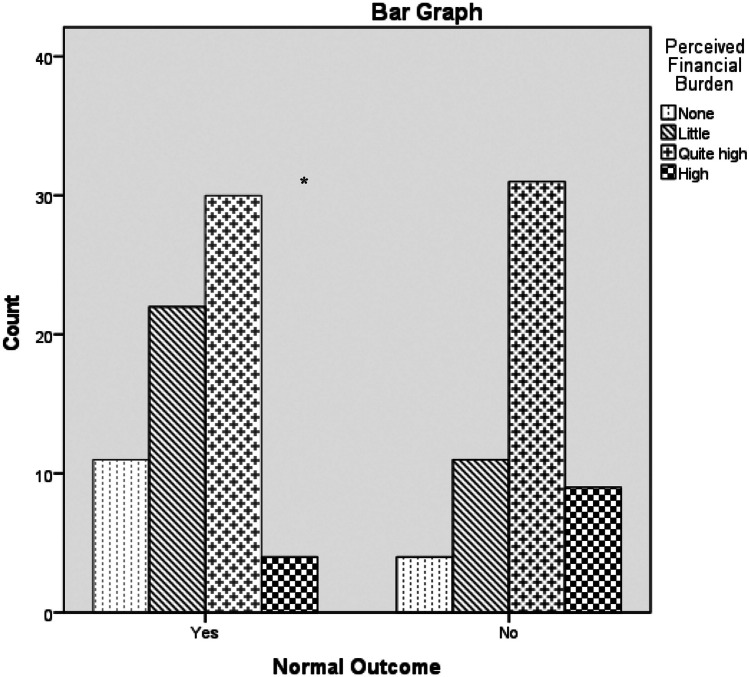
Comparison of perceived FB rates divided by normal versus adverse outcome

**Fig. 3:**
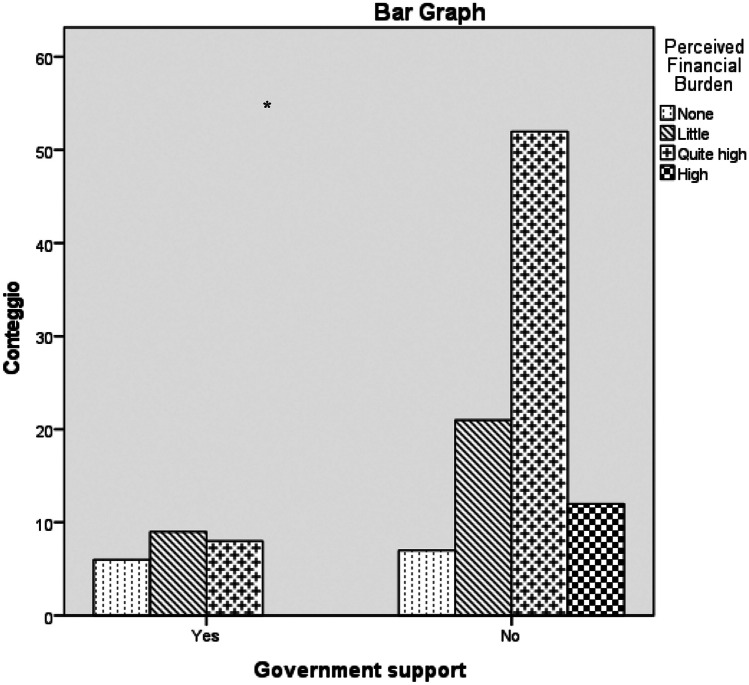
Comparison of perceived FB rates divided by presence versus absence of government financial support

Families having infants with adverse outcome experienced more hospitalization after NICU discharge (Cramer’s V = 0.264, χ^2^ = 8.853, *p* = 0.004) and higher rates of FB (Z =  −2.756, *p* = 0.006; Fig. 2). Significant high rates of perceived FB in family perceiving lack of support from the government were detected (Z =  − 3.419, *p* = 0.001; Fig. 3). We did not find any statistical difference comparing before and during COVID-19 and during and after COVID-19 pandemics with regard to private versus family pediatrician, costs of transportation at hospital and medical visits (*p* > 0.05),

Perceived FB correlated with costs of drugs (*ρ* = 0.271, *p* < 0.05), baby formula and dietary supplement (*ρ* = 0.385, *p* < 0.05). Infant adverse outcome (*ρ* = 0.251, *p* < 0.01) and comorbidities (*ρ* = 0.206, *p* < 0.05) were correlated with high rates of perceived FB. Among the infant developmental issues, presence of behavioral disorders (*ρ* = 0.186, *p* < 0.05) and language delay (*ρ* = 0.243, *p* < 0.01) were correlated with high rates of FB. Expenses linked to accompaniments at medical visits (*ρ* = 0.329, *p* < 0.01) and therapy sessions (*ρ* = 0.271, *p* < 0.05) correlated with higher FB rates. Lack of government support correlated with high FB perceived (*ρ* = 0.320, *p* < 0.01). Lack of government financial help was associated with the FB experienced by families (Exp(B) = 5.705, CI:[1.117,29.127], *p* < 0.05).

## Discussion

Our data show that the vast majority of the survey participants reported a significant impact of the birth of a premature child on the family finances.

### NICU hospitalization expenses

Our results provide a clearer scenario of sources that can influence costs related to NICU hospitalization leading to better identify specific profiles of families at risk to experience large amount of expenses during this period. In particular, families with extremely and very preterm infants have to face higher costs than parents of full-term babies. Considering birthweight, parents of babies with ELBW and VLBW face higher costs than parents of babies with adequate birthweight. This could be due to the severity and comorbidities associated with low gestational age and birthweight [12, 13] which can increase NICU length of stay. Our findings add knowledge to the financial costs related to infant clinical severity within a patient perspective framework. We recommend to screen all the families with preterm and low birthweight infants as increasing in infant length of stay could overwhelm parents’ capacity to face costs during NICU hospitalization. Detection and implementation of policies for families with overwhelming costs is important in order to reduce inequalities.

COVID-19 pandemic restrictions did not influence expenses faced by families during NICU hospitalization. This could be due to the fact that parents continued to visit NICU and asked for in person daily medical updates. Our sample also included families who were out of region during the preterm labor. They had dramatically high non-medical costs as they stayed in a hotel along the whole hospitalization. Preterm birth is frequently an unexpected event [14] that leads to drastic changes of family lives [15, 16]. NICU accommodations for parents could avoid cost transportations and improve their participation to the developmental care of their infant [17, 18].

Importantly, rate of NICU graduate mothers who quitted their job after childbirth was double as compared to mothers of healthy newborns in our country (18%) [19]. Our findings highlighted that mothers frequently left their job to take care of their infant in NICU while most fathers continued to work. During the hospitalization, 5% of mothers and 5% of fathers were also fired. Adverse childbirth and NICU hospitalization may be specific risk factors affecting the possibility for mothers to work. Those findings are particularly important as Italian mother unemployment rate is already one of the highest in Europe [20]. Policies need to take care of the multifaceted consequences of having a baby hospitalized in NICU as this stressful event may exacerbate parent unemployment especially of mothers.

Our study fills the gap regarding non-medical costs related to NICU hospitalization [3] and provide evidence of important side effects of the indirect costs (i.e., job loss) that influence family financial issues and disparities of parental experience during NICU hospitalization. In addition, our results provide a new perspective to further study factors influencing parental well-being, attachment to the baby and family experience during hospitalization [15, 21, 22]. Although all the families received at least one financial help from the government because of having a child, our findings prove that those helps are not sufficient to buffer the costs during NICU hospitalization and after discharge. Nowadays, policies do not consider neither NICU hospitalization nor its length of stay variability and their consequences on family income. Financial policies need to address direct non-medical and indirect costs faced by families and support them through the implementation of NICU accommodation services, ad hoc financial helps according to infant clinical condition and protect parents’ job.

We argue that baby hospitalization within NICU needs to be considered as stressful multifaceted event impacting parents both psychologically and financially. Those two aspects may affect each other. All the consequences due to NICU hospitalization deserve to be accounted in order to implement a Family-Centered Care that takes into account specific sources and needs of families.

### Expenses after NICU discharge

Almost all the families of NICU graduates experienced financial burden (FB) during the first years after NICU discharge. Among them, 60% experienced from moderate to high rates of FB. We highlighted that families of infants with adverse outcome experienced higher rates of FB as compared to families with typically developing infants. This could be due to the fact that parents of infants with developmental issues experience more frequent medical visits and hospitalizations as compared to parents with typically developing infants [23]. Those medical procedures include additional non-medical costs (i.e., transportations) and indirect costs (i.e., free-days from work that are superior to the ones established from the contract). Behavioral disorders and language delay were linked to high FB. As a result of the fact that psychomotor and speech therapy are not timely provided from the Italian National Health Service. Indeed, waiting lists for therapies can range from 2 to 48 months as confirmed from some families of our sample and websites of regional rehabilitation centers. The surveyed parents declared to spend median 300 euros for habilitation therapies per month when not provided from the National Health Service while transportation costs for therapies reached median 100 euros per month. Early intervention is essential to improve developmental trajectories of infants at risk for neurodevelopmental disorders [24]. Thus, National Health Service should timely match the needs for habilitation therapies of families in order to both foster infant neurodevelopmental trajectories and reduce the FB experienced by parents.

Parental perception of lack of economic helps from the government was the only factor affecting their FB. Importantly, feelings of being neglected from the National Health Service may furtherly exacerbate parental stress [25] leading them to be strictly focused on the disorder of their child [26] and impact the whole family unit [27]. Pathology exemption was one of the few sources of help for families. It is provided from the National Health System to all those patients that require specific services for the purpose of monitoring the evolution of the disease and its complications [28]. Pathology exemption buffers direct medical costs (e.g., developmental follow-up visits and clinical screening) for < 32 week preterm and/or with birthweight < 1500 g infants. Nevertheless, pathology exemption is valid up to the first 3 years of life of NICU graduates. This could influence FB of families as NICU graduates are at high-risk to develop neurocognitive issues across childhood [29–31].

Italian policies do not include any financial aid specific for family of NICU graduates infants and also maternity period do not match with the additional time that mothers and fathers need to stay close to the infant during hospitalization. On the contrary, other European countries approach to maternal and neonatal health differently. Some countries (e.g., Belgium, Austria, Germany) provide parents with additional free-from-work period starting since NICU discharge [32]. Financial aids are accorded to families whose NICU graduate infant develops neuro-motor abnormalities (e.g., cerebral palsy) homogenously across European countries [32]. However, prematurity itself as a risk condition, without any developmental issue, do not receive specific financial aids yet. Moreover, within countries differences emerge with regard to both family facilities and services (e.g., developmental follow-up).

Most parents indicated they needed help to manage family. Grandparents of NICU graduates were the most important source to both alleviate financial expenses and emotional support. Dependency from grandparents ranged from handling other children to having the family of NICU graduate infant living with them. Those findings underline the important supporting role of grandparents as well as the limited financial resources of a number of families after NICU discharge. Indeed, families could experience difficulties to achieve their own economic autonomy. Those findings reflect important difficulties related to achieve economic independence due to additional costs along with the ones usually needed for infants [33]. Policy makers should provide economic helps across the first years after discharge, in order to foster financial autonomy of families. With regards to other sources of help, surveyed families asked more frequently for nursery schools as compared to families of both Southern Italy (2%) and the whole Italian country (11%) [34]. Public nursery schools are not widely spread in Italy, especially in the Southern part of the country (ratio of public nursery schools and infants between 0 and 2 years is 15.2%) [34]. Thus, a large part of those families have to face financial costs such as private nursery schools with a cost that can reach 850 euros per month. In addition, families who needed nursery schools could be underestimated due to scarce presence of services and poor family income. This could lead parents to ask for baby sitters with costs ranging from 883.09 to 918.82 euros [35, 36].

The strong points of our study include the investigation of a broad array of costs and the relative FB both during NICU hospitalization and after discharge within the Italian context towards the lens of the patient perspective.

Among the limits, our survey included only infants who satisfied the Italian National Guidelines [37, 38] to be enrolled to the developmental follow-up of the preterm or at-risk of disability infants (i.e., preterm newborns with gestational age < 32 weeks and/or birthweight <1500 g and infants who suffered for hypoxic-ischemic encephalopathy). Thus, our study did not clarify the FB experienced by families of late preterm infants and > 1500 g at birth. Parents who were not fluent in Italian written form were also excluded leading to a lack in understanding financial burden of families coming from different countries.

The adoption of a societal perspective and the comparison of parents from different sociocultural backgrounds could enlarge the framework of financial costs of all the subjects involved within the NICU setting (i.e., the National Health Service, parents) and the type of costs faced (i.e., direct, non-direct medical costs and indirect costs).

## Conclusion

Our study provides new insights of financial burden experienced by Italian families of NICU graduates. This study highlighted sources (e.g., grandparents support) and difficulties (e.g., therapy costs) of NICU graduate families through the lens of patient perspective.

Financial policies that could be implemented include NICU accommodations to reduce the costs due to transportation and let parents be more present to care of their own infant. Tailored financial aids that take into considerations infant clinical severity, its consequent length of stay and needs after discharge are necessary. Policies should also protect both mother and father jobs during NICU hospitalization and provide extra out-of-work days due to infant health-related issues during the first years after discharge. Timely provided therapies and services are warranting to foster neurodevelopmental outcomes of NICU graduates as well as family management.

Our study promotes reflection on policies which should be adopted from the European governments that are similar to the Italian one in order to support NICU graduate families and reduce disparities.

### Supplementary Information

Below is the link to the electronic supplementary material.

Supplementary file1 (DOCX 21 KB)

## Data Availability

The datasets generated during and/or analysed during the current study are available from the corresponding author on request.

## Abbreviations

ELBW: Extremely low birthweight
FB: Financial burden
LBW: Low birthweight
NICU: Neonatal intensive care unit
