# Supplementary material for: The financial burden experienced by families during NICU hospitalization and after discharge: A single center, survey-based study
Source: Eur J Pediatr. 2023 Dec 1;183(2):903–13. doi: 10.1007/s00431-023-05352-y (PMC10912114; doi:10.1007/s00431-023-05352-y)
Supplement: Supplementary file 1 — Supplementary file1 (DOCX 21 KB) [file 431_2023_5352_MOESM1_ESM.docx]

**Supplementary material**

Study partecipation consent

- Yes
- No

**I. Parents data and family environment:**

1. Maternal age:

_________________

1. Maternal Education

- Primary school
- Middle school
- High school
- University
- Other

1. Mother’s occupation

___________________________

1. Maternal Nationality

- Italian
- Foreigner

1. Paternal age

_________________________

1. Paternal education

- Primary school
- Middle school
- High school
- University
- Other

1. Paternal Nationality

- Italian
- Foreigner

1. Paternal occupation

__________________________________

1. Place of residence and district

__________________________________

1. Number of family members. Please specify the degree of kinship

__________________________________

1. Other children

- Yes
- No

1. If other children are present, please specify sex and age

___________________________________

1. You are living in

- Owned house
- House for rent
- Parents’ house
- Other ____________

**II. Characteristics of the infant admitted in the NICU and expenses during hospitalization**

1. Date of birth of the infant admitted to the NICU

________________________

1. Hospital where infant was admitted

________________________

1. Type of pregnancy
   - Spontaneous pregnancy
   - Assisted fertilization
   - Other: ______________
2. Twin delivery

- Yes
- No

1. If Yes, please specify number of twins:

_________________

1. Gestational of the infant admitted to the NICU

_________________

1. Birthweight of the infant admitted to the NICU

__________________

1. Sex of the infant(s) admitted to the NICU

- Female
- Male
- Male and female
- Males
- Females

1. Diagnosis at the NICU admission (you can pick all the applicable answers)

- Prematurity
- Low birthweight
- Twinning
- Respiratory distress
- Hypoxic-ischemic encephalopathy
- Sespis (infection)
- I do not know
- Other: _____________

1. Length of stay in the NICU (number of days)

_____________________________

1. What transport did you use to visit your child in the NICU?

- Own conveyance
- Public transport
- Rented car
- Taxi
- Other: _________

**III. Infant health and development after NICU discharge**

1. What kinds of outcomes did your child have after NICU hospitalization? (you can pick all the applicable answers)

- Typical development
- Broncho dysplasia
- ROP (visual problems)
- NEC (intestinal problems)
- Post-surgical short intestine
- Language delay
- Behavioral disorder
- Intellectual disability
- Global delay of the psychomotor development
- Anomalies of muscular tone (hypotonia, hypertonia, clumsiness)
- Cerebral palsy
- Autism
- Feeding problems
- Growth deficit
- Deafness
- Blindness
- Heart disease
- Other: __________

1. Did your child experience other hospitalizations after NICU discharge?

- Yes
- No

1. If Yes, please specify the reason of the hospitalization and its length

_____________________________________

1. Did your child need day hospital?

- No
- Yes
- Yes, within our hospital

1. If Yes, please specify the reason

_______________________________________

1. Did your child need medical outpatient visits after NICU discharge?

- No
- Yes
- Yes, within our hospital

1. If Yes, please specify the reason

____________________________________

1. Do you have exemption due to your income?

- Yes
- No

1. Do you have exemption due to the disease of your child?

- Yes
- No

1. If Yes, please specify the disease of your child

________________________________________________________

1. Are you receiving an economic aid due to the pathology of your child?

- Yes
- No

1. If Yes, does it cover the expenses?

- No
- Partially
- Completely

**IV. Therapies and their related expenses**

1. Does your child need special aids? (you can pick all the applicable answers)

- No
- Wheelchair
- Prosthesis
- Glasses
- Assisted ventilation
- High-flow nasal cannula
- Pulse oximeter
- PEG
- Nasogastric tube
- Other: _____________

1. Do you have to face expenses due to your child’s special aids?

- Yes
- No

1. If yes, please specify monthly cost for special aids

____________________________________________

1. Could you please specify costs due to laboratory exams?

_____________________________________________

1. Does your child need physiotherapy?

- Yes
- No

1. If Yes, could you specify the age of beginning?

________________________________

1. Could you please specify costs due to physiotherapy if not provided from the National Health Service?

_________________________________

1. Does your child need habilitation therapy?

- Yes
- No

1. Types of habilitation therapy needed (you can pick all the applicable answers)

- Speech therapy
- Psychomotor therapy
- Respiratory physiotherapy
- Other: ______________

1. If Yes, could you specify the age of beginning?

__________________________________________

1. Could you please specify costs due to habilitation therapy if not provided from the National Health Service?

___________________________________________

1. Could you please specify costs due to transportation of your child at habilitation therapy sessions?

____________________________________________

1. Does your child need home nursing care?
   - Yes
   - No
2. If Yes, please provide the age of beginning

___________________________________

1. Could you please specify costs due to home nursing care if not provided from the National Health Service?

____________________________________

**V. Specific expenses and clinical visits**

1. Could you please specify, if present, monthly cost due to vitamins?

________________________________________

1. Could you please specify, if present, monthly cost due to drugs?

________________________________________

1. Could you please specify, if present, monthly cost due to milk and other supply?

_________________________________________

1. Does your child have clinical check-ups with family pediatrician with the private one or both? (you can pick all the applicable answers)

- Family pediatrician
- Private pediatrician

1. Does your child have monthly expenses for clinical visits?

_________________________________________

1. How many times do you bring your child to the hospitals for clinical check-ups?

- It does not happen
- Once a year
- Twice a year
- More than three times a year

1. What transportation do you use to bring your child to the hospitals for clinical check-ups?
   - Public transport
   - Own conveyance
   - Rented car
   - Taxi
   - Other: ___________
2. How much cost bringing your child to the hospitals?

_________________________________________

**VI. Occupational changes during NICU hospitalization and work leaves after discharge**

1. Did mother have to leave her job during NICU hospitalization?

- Yes
- No
- Other: ___________

1. Did father have to leave his job during NICU hospitalization?

- Yes
- No
- Other: ___________

1. Did mother have been fired during NICU hospitalization?

- Yes
- No
- Other: ________

1. Did father have been fired during NICU hospitalization??

- Yes
- No
- Other: ________

1. How many work leaves do you need due to management of your child within one year?

- Less than one month
- One month
- More than one month
- Other: ___________

1. Are work leaves more than the ones established from your contract?

- Yes
- No

1. How many unpaid work leaves had been within one year?

___________________________________________

**VII. Support and burden of the NICU experience**

1. What persons did you need to handle family management?
   - Grandparents
   - Baby sitter
   - Domestic worker
   - Nursery school
   - No one
   - Other: ___________
2. Did you feel supported from your family relatives?

- Yes
- No

1. If Yes, what kinds of support did you receive? (you can pick all the applicable answers)

- Economic aid
- Transportations
- House management
- Other children management
- Other: ______________

1. Did you feel supported from the government?

- Yes
- No

1. Did you receive economic aids when your child was staying in the NICU? (you can pick all the applicable answers)

- No
- Single check
- Baby bonus
- “Mom tomorrow” bonus
- Nursery school bonus
- “Third child” bonus
- Other: __________________

1. If Yes, did they fulfill your expenses?

- Completely
- Partially
- No

1. Did NICU hospitalization and management after discharge have had a financial burden difficult to sustain?

- Not at all
- Little
- Moderate
- High
